# Comparative sedimentation equilibrium analysis of two IgG1 glycoforms: IgGCri and IgGWid

**DOI:** 10.1007/s00249-023-01656-x

**Published:** 2023-05-17

**Authors:** Khalil Abu Hammad, Vlad Dinu, Thomas E. MacCalman, Jacob Pattem, Margaret Goodall, Richard B. Gillis, Roy Jefferis, Stephen E. Harding

**Affiliations:** 1 National Centre for Macromolecular Hydrodynamics (NCMH), School of Biosciences, University of Nottingham, Sutton Bonington, LE12 5RD UK; 2 School of Biosciences, University of Nottingham, Sutton Bonington, LE12 5RD UK; 3 Institute of Immunology & Immunotherapy, College of Medical & Dental Sciences, University of Birmingham, Birmingham, B15 2TT UK; 4 College of Business, Technology and Engineering, Food and Nutrition Group, Sheffield Hallam University, Sheffield, S1 1WB UK

**Keywords:** SEDFIT-MSTAR, MULTISIG, Fc/Fab glycans, c(*M*)-distribution, c(*s*)-distribution

## Abstract

The solution properties of two different glycoforms of IgG1 (IgG1Cri and IgG1Wid) are compared using primarily sedimentation equilibrium analysis with two complementary analysis routines: SEDFIT-MSTAR and MULTISIG. IgGCri bears diantennary complex-type glycans on its Fc domain that are fully core fucosylated and partially sialylated, whilst on IgGWid, they are non-fucosylated, partially galactosylated and non-sialylated. IgGWid is also Fab glycosylated. Despite these differences, SEDFIT-MSTAR analysis shows similar weight average molar masses *M*_w_ of ~ (150 ± 5) kDa for IgGCri and ~ (154 ± 5) kDa for IgGWid and both glycoforms show evidence of the presence of a small fraction of dimer confirmed by MULTISIG analysis and also by sedimentation coefficient distributions from supportive sedimentation velocity measurements. The closeness of the sedimentation equilibrium behaviour and sedimentation coefficient distributions with a main peak sedimentation coefficient of ~ 6.4S for both glycoforms at different concentrations suggest that the different glycosylation profiles do not significantly impact on molar mass (molecular weight) nor conformation in solution.

## Introduction

In the last 3 decades since the advent of a new generation of commercial analytical ultracentrifuges with automatic data capture and analysis procedures, the technique of sedimentation equilibrium in the analytical ultracentrifuge seems to have taken somewhat a back stage compared to sedimentation velocity. For molar mass analysis, the fundamental disadvantage of the latter’s dependence on conformational/frictional effects has been mostly overcome by data fitting to yield an estimate for the translational diffusion coefficient, which when combined with the sedimentation coefficient provides an estimate, particularly for ideal systems, of the molar mass using the Svedberg equation (see, e.g., Tanford 1961). Sedimentation equilibrium remains, however, as a complementary probe, and in parallel with sedimentation velocity, there have been important advances which also take advantage of on-line data capture, such as SEDFIT-MSTAR (Schuck et al 2014), which has considerably extended the capability of an earlier procedure developed in the 1980s (Creeth and Harding 1982). Another relatively recent development has been the MULTISIG (Gillis et al 2013) algorithm which models the solute distribution at sedimentation equilibrium in terms of a large number (17) of discrete component fits from which a discrete or quasi-continuous molar mass (molecular weight) distribution can be obtained depending on what system is being represented.

Both analyses when used together—and in conjunction with sedimentation velocity—are particularly useful for the characterisation of glycosylated systems because of their greater polydispersity or heterogeneity compared to proteins (see Beck et al. 2019).

**Fig. 1:**
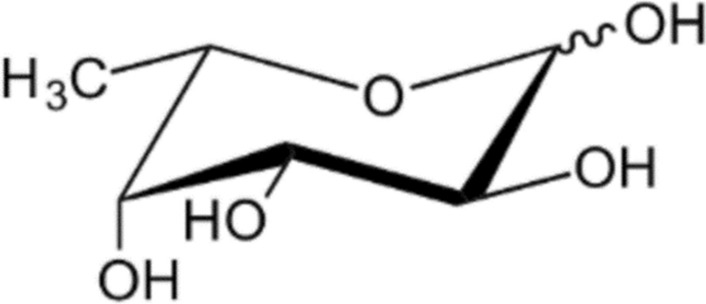
Fucose with its characteristic hydrophobic methyl group. Different fucosylations can have a profound effect on efficacy and stability. Courtesy of Chris Lawson, Carbosynth Ltd

In an earlier study, Mimura and co-workers (Mimura et al. 2007) reported the glycoform profiles of two human IgG1 subclass myeloma para-proteins, IgG1Cri and IgG1Wid, and demonstrated that they exhibited differing glycoform profiles. Electrospray ionization mass spectroscopic analysis of the microheterogeneity of the Fc fragments showed that they differed in the diantennary complex-type glycan structures N-linked at asparagine residue 297 (Asn 297). Glycoprotein IgG1Cri bears a glycan that is fully core fucosylated with a proportion bearing a terminal sialic acid sugar residue; in contrast, the IgG1Wid protein bears a non-fucosylated glycan, and is only partially galactosylated and devoid of sialylation. In addition, the Fab fragment of IgG1Wid was shown also to bear glycan within the heavy-chain variable region; a structural feature that is found within 15–20% of normal polyclonal IgG preparations (Mimura et al. 2021). The presence or absence of fucosylated oligosaccharides within the Fc region of IgG has been shown to influence the activation of downstream clearance mechanisms resulting in the killing/clearance of IgG immune complexes (Mimura et al. 2022). Consequently, IgG1 antibody therapeutics are engineered to selectively bear core fucose residues (Fig. 1) or to be devoid of this sugar.

The mass spectroscopy value for the molar mass of both IgG1s is similar ~ 150,000 g/mol. The question is does this translate also to similar properties in solution and is there evidence for any significant association or aggregation effects? We investigate using a combination of sedimentation equilibrium with sedimentation velocity in the ultracentrifuge.

## Materials and methods

### Materials

The monoclonal IgG1 immunoglobulins Wid and Cri had been isolated and purified from myeloma sera as previously reported (Radcliffe et al. 2007; Mimura et al 2007, 2021). They had been stored at − 20.0 °C and then thawed and dialysed into a phosphate-chloride buffer pH 7.0 made up to an ionic strength *I* = 0.10 M according to Green (1933). 1 L of buffer contained 4.595 g Na_2_HPO_4_.12H_2_O and 1.561 g KH_2_PO_4_ with 2.923 g NaCl added.

### Sedimentation equilibrium in the analytical ultracentrifuge

Sedimentation equilibrium experiments were performed in a Beckman XL-I analytical ultracentrifuge (AUC) equipped with Rayleigh interference optics, as previously described (see, for example, Heinze et al. 2011; Nikolajski et al. 2014). 12 mm optical path double sector cells with an epoxy centrepiece and sapphire windows in an aluminium housing were employed: solution and solvent (buffer) reference channels were injected with a volume of 100 μL. Solutions were run at 12,000 rpm at 20.0 °C. A low loading concentration of 0.5 mg/mL was chosen so as to minimize the effects of thermodynamic non-ideality (co-exclusion phenomena). Scans with Rayleigh interference optics were taken every hour until equilibrium was reached. Values for the partial specific volume $\overline{v}$ of 0.729 mL/g for IgGCri, and 0.728 mL/g for IgGWid were determined from compositional analysis (Durschlag and Zipper 1994). Two software analysis routines were employed. Plots of point weight average molar mass *M*_w_(*r*) as a function of radial displacement *r* from the centre of rotation were produced using SEDFIT-MSTAR (Schuck et al 2014). MULTISIG (Gillis et al. 2013) was also run using its standard 17 component system with 20 iterations for each concentration yielding molar mass distributions, c(*M*) vs *M*_w_.

### Sedimentation velocity in the analytical ultracentrifuge

Sedimentation coefficient distributions c(*s*) vs the sedimentation coefficient *s* were also determined using a Beckman XL-I analytical ultracentrifuge equipped with Rayleigh interference optics. 12 mm optical path double sector cells with an epoxy centrepiece and sapphire windows in an aluminium housing were also employed, and solution and solvent (buffer) reference channels were filled to 400 μL. A rotor speed of 49,000 rpm at a temperature of 20.0 °C was used. Analysis was carried out using SEDFIT (Schuck 2003; Dam and Schuck 2004) which gives an apparent distribution of (diffusion-corrected) sedimentation coefficient c*(s)* vs *s* and the corresponding (apparent) weight average sedimentation coefficient, *s*. We use a scan range from 0.1 to 15S, with regularisation (confidence F ratio) 0.95 and resolution set to 250 and the data points were fitted using B-splines (these do not affect the position of the peaks). Loading concentrations of 0.5 and 1.0 mg/ml were employed for each immunoglobulin. After correction for average radial dilution these translate to actual sedimenting concentrations of ~ 0.4 and 0.8 mg/mL, facilitating comparisons. All sedimentation coefficients were normalised to standard solvent conditions (the viscosity and density of water at 20.0 °C).

## Results and discussion

### Molar mass distributions

**Fig. 2:**
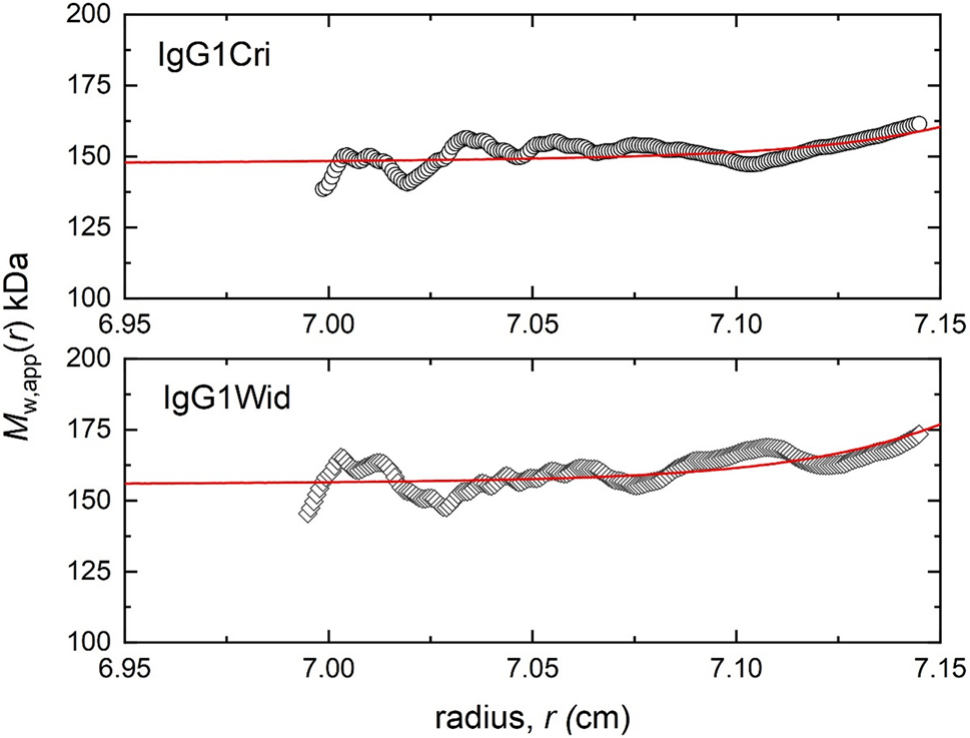
SEDFIT-MSTAR output showing the point or local apparent weight average molar masses *M*_*w*_(*r*) of IgG1Cri and IgG1Wid (at loading concentrations of 0.5 mg/mL) as a function of radial position *r*; red line is fit

Figure 2 compares distributions of the point weight average molar mass *M*_w_(*r*) vs radial displacement *r* obtained from SEDFIT-MSTAR (Schuck et al 2014) for IgG1Cri (top) and IgG1Wid (bottom), obtained at a loading concentration *c* of 0.5 mg/mL: very similar profiles were obtained and similar values for *M*_w_(*r*) at the meniscus (*r* = 6.91 cm) were obtained, *M*_w_ ~ (150 ± 5) kDa for IgGCri and ~ (154 ± 5) kDa for IgGWid (error estimates are from the fit). Both glycoforms show an increase in *M*_w_(r) towards the cell base to ~ 170–175 kDa, clearly the result of the presence of some aggregation/association product.

**Fig. 3:**
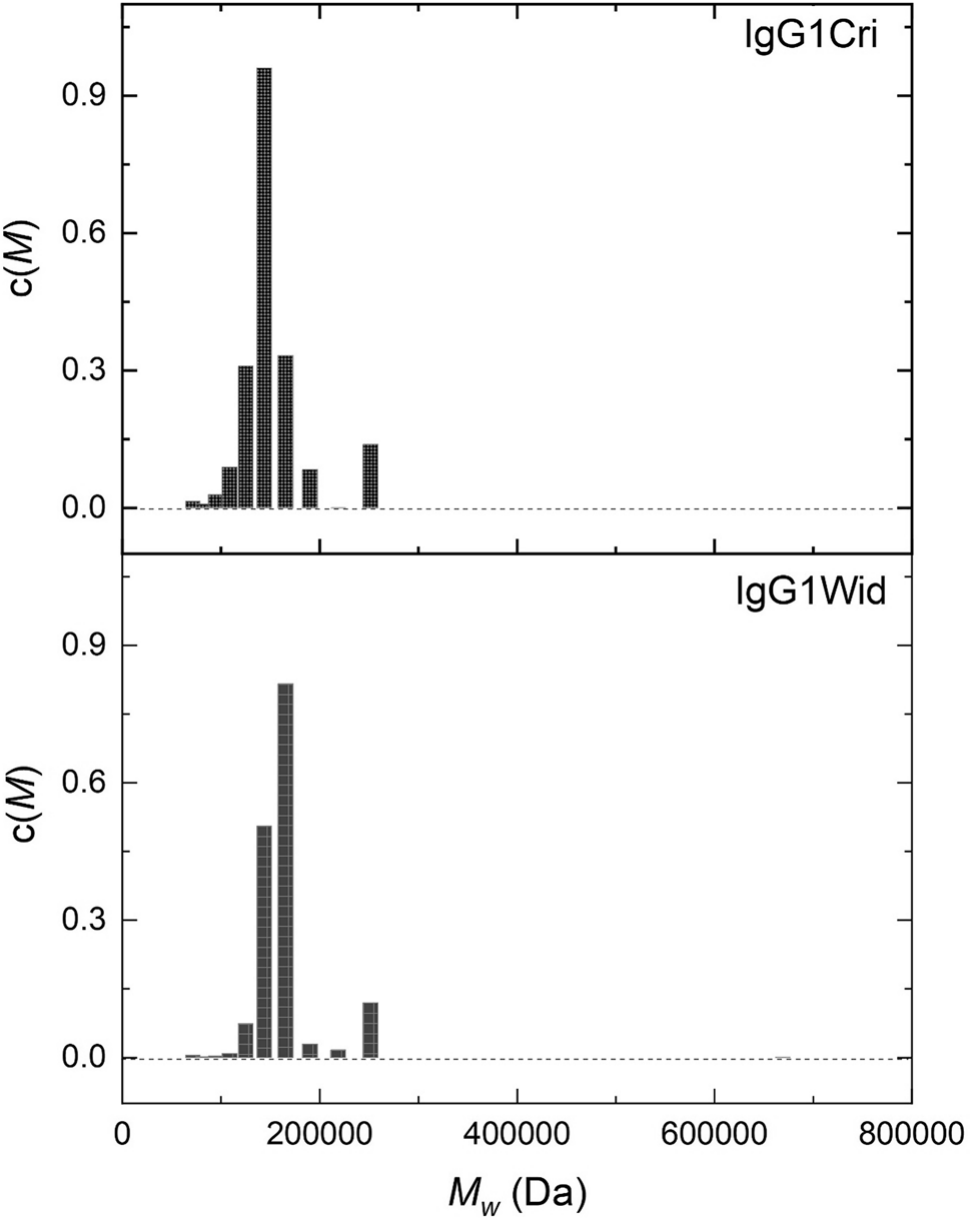
Sedimentation equilibrium analysis showing the MULTISIG analysis yielding the weight average molar mass distribution c(*M*) vs (*M*_w_, Da) for IgG1Cri and IgG1Wid (at loading concentrations of 0.5 mg/mL)

These observations are reinforced by MULTISIG (Gillis et al 2013) analysis of c(*M*) vs *M*_w_ (Fig. 3), which show better the presence of an association product and which is most likely a dimer.

### Sedimentation coefficient distributions

**Fig. 4:**
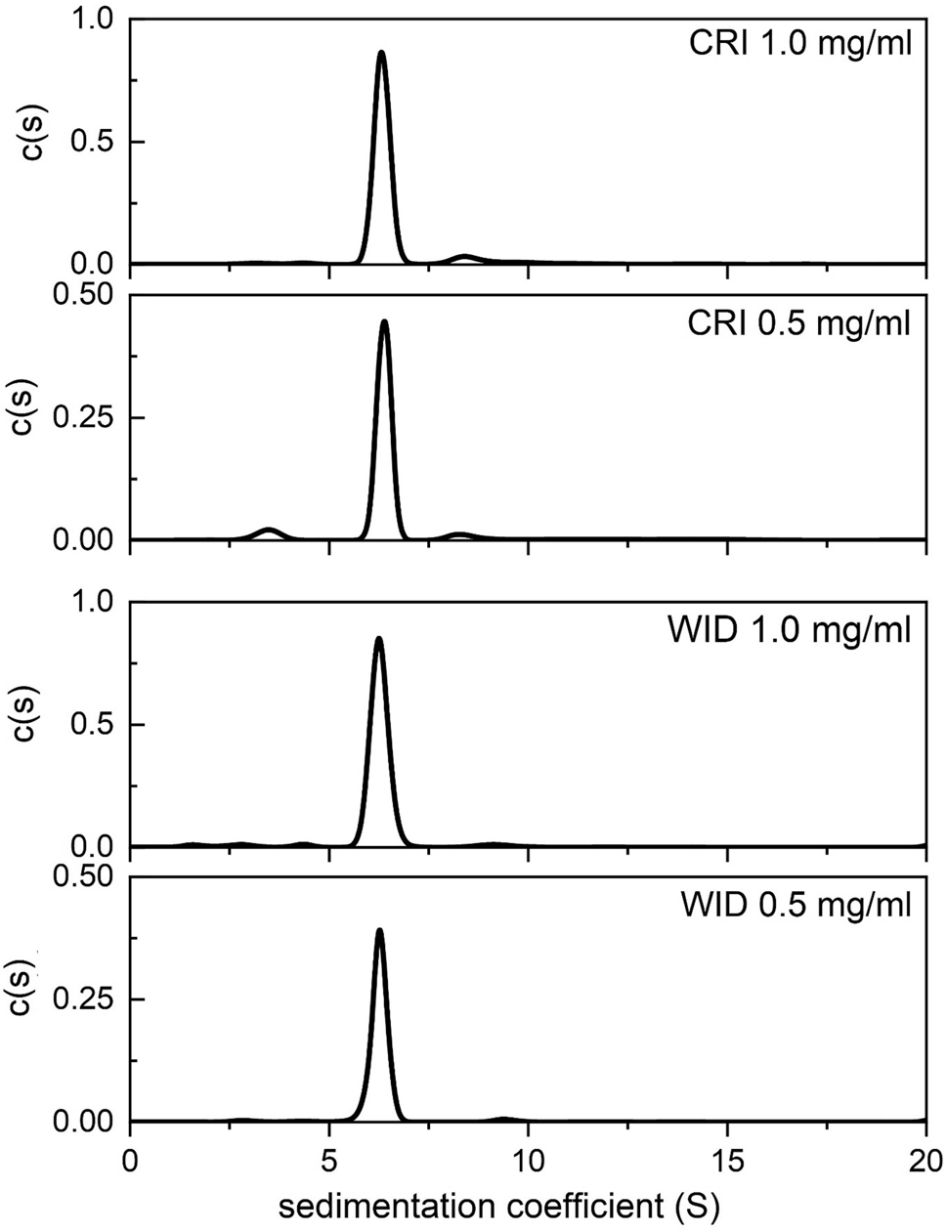
Comparative sedimentation coefficient distributions c(*s*) vs *s* of IgG1Cri and IgG1Wid at loading concentrations of 0.5 and 1.0 mg/mL (corresponding to average sedimenting concentrations of 0.4 and 0.8 mg/mL, respectively). The c(*s*) axes have been normalised for the different cell loading concentrations

This observation is further reinforced by the sedimentation coefficient distributions, c(*s*) vs *s*, obtained using SEDFIT and at two loading concentrations 0.5 and 1.0 mg/mL (Fig. 4). IgG1Cri and IgG1Wid both sediment with near-identical rate with sedimentation coefficients of ~ 6.4S, and both show the presence of dimer, sedimenting at ~ 8.5S for IgGCri and ~ 9.5S for IgGWid. The predicted value based on a spherical particle assumption for the monomer and similar conformation for the dimer is ~ 10S (based on the scaling or power law for spheres of *s* $\propto$ *M*^2/3^). NB the agreement holds for non-spherical particles where monomer and dimer have the same conformation or translational frictional ratio. The slight difference with prediction may be due to deviations from this assumption for both monomer and dimer, i.e., the dimers may adopt a conformation that has a significantly higher frictional ratio than the monomer. The monomer and dimer may also be in a rapid dynamic equilibrium within the higher *s* value peak, which would also cause a lowering of the expected dimer *s *value (see Harding and Rowe 2010).

Both glycoforms show also the presence of lower molar mass material, consistent with the lower molar mass tail on the MULTISIG plots, more pronounced for IgGCri. This may be a degradation product but is more likely to be due to trace residual contaminants left over from isolation and purification from the myeloma sera (Mimura et al 2007).

### Concluding remarks

Despite being different glycoforms of IgG1, SEDFIT-MSTAR analysis shows similar weight average molar masses *M*_w_ of ~ (150 ± 5) kDa for IgGCri and ~ (154 ± 5) kDa for IgGWid and both show evidence of the presence of a small fraction of dimer confirmed by MULTISIG analysis and also by sedimentation coefficient distributions from supportive sedimentation velocity measurements. Because of the presence of these discrete aggregates, the employment of standard M* extrapolation to the cell base to estimate the whole distribution molar masses (Creeth and Harding 1982) was not useful in this case: so, the use of point average molar masses versus radial displacement—also obtained from SEDFIT-MSTAR (Schuck et al 2014)—was used. The closeness of the sedimentation equilibrium behaviour and sedimentation velocity sedimentation coefficient distributions from SEDFIT with a main peak sedimentation coefficient of ~ 6.4S for both glycoforms at different concentrations suggest that the different glycosylation profiles do not significantly impact on molar mass nor conformation in solution, at least for this pair of IgG1 antibodies.

In the current context, “heterogeneity” or “microheterogeneity” due to variations in glycoform profiles—as assessed previously using electrospray ionization mass spectroscopic analysis (Mimura et al 2007)—provided the platform for this short hydrodynamic-based study. Had that platform not been available it may have been possible to have studied this using the Bayesian tools available in SEDFIT (see Brown et al 2007). Whatever, it should be borne in mind that in a wider context, the natural “aging” of a protein in vivo results from changes to amino acid side chain structures, e.g., oxidation of methionine; deamidation of asparagine/glutamine residues; disulphide exchange, clipping of C-terminal residues, cyclisation of N-terminal glutamic acid residues, etc. Such natural structural changes may be amplified when purifying any protein from blood/serum/plasma or of recombinant protein when produced ex vivo, i.e., from cell culture supernatant. It is well known that the biopharmaceutical industry is much exercised by such issues: in the development of a possible mAb therapeutic, any candidate having susceptible amino acids within the complementarity determining regions (CDRs) is rejected.

The different sedimentation coefficients of the higher molar mass components (~ 8.5S for IgG1Cri and ~ 9.5S for IgG1Wid)—if they correspond to dimer—might correspond to different conformations. To analyse this further would require combination with other hydrodynamic and X-ray scattering techniques to address uniqueness and (time average) hydration singularities using the “Crystallohydrodynamics approach” (Carrasco et al 2001; Longman et al 2003; Harding et al 2004; Lu et al 2006, 2007).

## Data Availability

Raw data are available for the corresponding authors.

## References

[CR1] Beck A, Liu H (2019). Macro- and micro-heterogeneity of natural and recombinant IgG antibodies. Antibodies.

[CR2] Brown PH, Balbo A, Schuck P (2007). Using prior knowledge in the determination of macromolecular size-distributions by analytical ultracentrifugation. Biomacromol.

[CR3] Carrasco B, Garcia de la Torre J, Davis KG, Jones S, Athwal D, Walters C, Burton DR, Harding SE (2001). Crystallohydrodynamics for solving the hydration problem for multi-domain proteins: open physiological conformations for human IgG. Biophys Chem.

[CR4] Creeth JM, Harding SE (1982). Some observations on a new type of point average molecular weight. J Biochem Biophys Meth.

[CR5] Dam J, Schuck P (2004). Calculating sedimentation coefficient distributions by direct modeling of sedimentation velocity concentration profiles.

[CR6] Durchschlag H, Zipper P (1994). Calculation of the partial volume of organic compounds and polymers. Prog Colloid Polym Sci.

[CR7] Gillis RB, Adams GG, Heinze T, Nikolajski M, Harding SE, Rowe AJ (2013). MULTISIG: a new high-precision approach to the analysis of complex biomolecular systems. Eur Biophys J.

[CR8] Green AA (1933). The preparation of acetate and phosphate buffer solutions of known pH and ionic strength. J Amer Chem Soc.

[CR9] Harding SE, Rowe AJ (2010). Insiight into protein-protein interactions from analytical ultracentrifugation. Biochem Soc Trans.

[CR10] Harding SE, Longman E, Ortega A, Kreusel K, Tendler SB, King K, Garcia de la Torre J (2004). Use of the sedimentation coefficient for modelling antibodies. Refinements to the Crystallohydrodynamics approach. Prog Coll Polym Sci.

[CR11] Heinze T, Nikolajski M, Daus S, Besong TMD, Michaelis N, Berlin P, Morris GA, Rowe AJ, Harding SE (2011). Protein-like oligomerisation of carbohydrates. Angewand Chem Int Ed.

[CR12] Longman E, Kreusel K, Tendler SJB, Fiebrig I, King K, Adair J, O'Shea P, Ortega A, Garcia de la Torre J, Harding SE (2003). Estimating domain orientation of two human antibody IgG4 chimeras by crystallohydrodynamics. Europ Biophys J.

[CR13] Lu Y, Longman E, Davis KG, Ortega A, Grossmann JG, Michaelsen TE, Garcia de la Torre JG, Harding SE (2006). Crystallohydrodynamics of protein assemblies: combining sedimentation, viscometry, and x-ray scattering. Biophys J.

[CR14] Lu Y, Harding SE, Michaelsen TE, Longman E, Davis KG, Ortega A, Grossmann JG, Sandlie I, Garcia de la Torre J (2007). Solution conformation of wild type and mutant IgG3 and IgG4 immunoglobulins using Crystallohydrodynamics: possible implications for complement activation. Biophys J.

[CR15] Mimura Y, Ashton PR, Takahashi N, Harvey DJ, Jefferis R (2007). Contrasting glycosylation profiles between Fab and Fc of a human IgG protein studied by electrospray ionization mass spectrometry. J Immunol Methods.

[CR16] Mimura Y, Saldova R, Mimura-Kimura Y, Rudd PM, Jefferis R (2021). Micro-heterogeneity of antibody molecules. Exp Suppl.

[CR17] Mimura Y, Mimura-Kimura Y, Saldova R, Rudd PM, Jefferis R (2022). Enhanced immunomodulatory effect of intravenous immunoglobulin by Fc galactosylation and nonfucosylation. Front Immunol.

[CR18] Nikolajski M, Adams GG, Gillis R, Besong DT, Rowe AJ, Heinze T, Harding SE (2014). Protein-like fully reversible tetramerisation and super-association of an aminocellulose. Sci Rep.

[CR19] Radcliffe CM, Arnold JN, Suter DM, Wormald MR, Harvey DJ, Royle L (2007). (2007) Human follicular lymphoma cells contain oligomannose glycans in the antigen-binding site of the B-cell receptor. J Biol Chem.

[CR20] Roark DE, Yphantis DA (1969). Studies of self-associating systems by equilibrium ultracentrifugation. Ann NY Acad Sci.

[CR21] Schuck P (2003). On the analysis of protein self-association by sedimentation velocity analytical ultracentrifugation. Anal Biochem.

[CR22] Schuck P, Gillis RB, Besong TM, Almutairi F, Adams GG, Rowe AJ, Harding SE (2014). SEDFIT–MSTAR: molecular weight and molecular weight distribution analysis of polymers by sedimentation equilibrium in the ultracentrifuge. Analyst.

[CR23] Tanford CA (1961). Physical Chemistry of Macromolecules.

